# Implications of federalism: patterns of healthcare utilisation across Austrian federal states – insights from the Health Interview Surveys

**DOI:** 10.3389/fpubh.2026.1796254

**Published:** 2026-07-08

**Authors:** Roland Kraxner, Thomas E. Dorner, Dominik Roth, Kathryn Hoffmann

**Affiliations:** 1 Department of Primary Care Medicine, Center for Public Health, Medical University of Vienna, Vienna, Austria; 2 Academy for Ageing Research, Haus der Barmherzigkeit, Vienna, Austria; 3 Department of Social and Preventive Medicine, Center for Public Health, Medical University of Vienna, Vienna, Austria; 4 Department of Emergency Medicine, Medical University of Vienna, Vienna, Austria

**Keywords:** Austria, delivery of health care, health policy, health services accessibility, survey

## Abstract

**Background:**

Austria’s nine federal states are responsible for healthcare provision, particularly hospital services. However, differences in political arrangements between states have resulted in spatial disparities in healthcare. This study evaluated whether the utilisation of primary and secondary healthcare services showed regional differences over time.

**Methods:**

This analysis was based on data from the most recent Austrian Health Interview Survey (ATHIS) series conducted in 2014 and 2019, with a total of 31,231 participants. Data on physician density and population numbers were retrieved from the Austrian Medical Chamber and Statistics Austria. Healthcare utilisation behaviour was analysed using descriptive statistics, correlation analysis and multivariable logistic regression models.

**Results:**

Highly urbanised regions showed higher rates of ambulatory specialist visits compared with less urbanised regions but lower visit counts of general practitioners (GPs). The reversed utilisation trend was observed for regions of low urbanisation (ambulatory specialist visits: OR 0.79, 95% CI 0.72–0.86, *p* < 0.01, for regions of low urbanisation). Hence, health service utilisation varied widely across federal states. Overall, physician density in the federal states increased for specialists (i.e., from one specialist per 873 individuals in 2014 to one per 824 in 2019), whereas the density of GPs decreased. Specialist density was correlated with patterns of increasing specialist utilisation (Spearman’s rho correlation coefficient: −0.69, *p* = 0.04).

**Conclusion:**

Healthcare utilisation varied according to the degree of urbanisation. The increasing utilisation of specialised care is associated with specialist density. These findings should be emphasised in the context of current health reforms in Austria, especially regarding primary care.

## Introduction

Health services worldwide are facing major challenges due to ageing populations and the global phenomena of increases in non-communicable diseases, such as diabetes mellitus and obesity, as well as the reoccurrence of communicable diseases, such as SARS-CoV-2. In addition, the global shortage of healthcare professionals puts pressure on the currently limited healthcare workforce (1). Therefore the proper allocation of financial and human resources to primary (i.e., general practitioners [GPs] and registered nurses working in practices and primary healthcare units) and secondary (i.e., in- and outpatient hospital services and ambulatory specialist practices) care services is of utmost importance. Primary care systems can address the majority of a population’s health issues through the provision of preventive and curative care (2, 3). Numerous studies have shown that continuity of care reduces hospitalisation rates, reduces increases in healthcare costs and leads to higher patient satisfaction (4–6).

Access to healthcare varies across Europe, depending on national policies. In Austria, the utilisation of health services is unregulated; that is, almost all levels of care can be freely accessed. Several studies have shown that all levels of care are highly utilised in Austria, especially secondary care (7–14). While statutory health insurance (SHI) funds cover all public health service visits for patients in Austria, the organisation and financing of healthcare services in Austria are divided between the national level, SHI funds and the respective federal states. In particular, SHI funds primarily cover so-called extramural ambulatory care, which refers to ambulatory practices outside of hospitals, while federal states are largely responsible for providing state hospital care. However, healthcare legislation is mainly conducted at the national level. Any changes in patient flow would impact the financial burden shared between these bodies. SHI is mandatory for all Austrians, but patients may also seek care from private in- and outpatient health providers. These services must be paid out of pocket but may be reimbursed by private health insurances. These are voluntary health insurances (VHI), which offer a variety of additional services, such as single hospital bedrooms; however, they also cover fees for physicians without SHI contracts. VHI are available without eligibility restrictions, provided premiums are paid. More than one third of the Austrian population is covered by VHI in addition to their mandatory SHI. The main reasons for choosing VHI include access to better medical care and covering gaps of the SHI system (14).

As long as they comply with national laws, Austrian federal states have the authority to control large parts of the Austrian healthcare system, especially organisational structures in the hospital sector (9, 15). Therefore, the utilisation of healthcare services may vary according to the respective federal states’ demographic structures and regional policies. Previous studies have demonstrated significant variations among the nine Austrian federal states in terms of cardiovascular death rates, associated health status and health behaviour (16, 17). Moreover, physician density (both with and without SHI contracts) varies widely among different federal states due to many regional factors, including socio-economic characteristics (18). In densely populated regions like Vienna, the only metropolitan region of Austria, with around 2 million inhabitants, there are more than 7 doctors per 1,000 residents; in predominantly rural regions, such as the federal state of Upper Austria, there are 4.5–4.8 physicians per 1,000 inhabitants as of 31 December 2024 (19).

Health indicators in Austria, such as satisfaction with healthcare, medication intake and utilisation of healthcare services, are evaluated every 5–8 years through extensive population-based surveys commissioned at the national level, the Austrian Health Interview Surveys (ATHIS). To date this survey series consists of data from the years 2006/07, 2014 and 2019 (20–22). The utilisation of specialist services in ambulatory practices was evaluated at the federal state level using ATHIS 2006/07 data. One key finding was the large variation in ambulatory specialist visits (either directly or with a concomitant GP visit) across the federal states (9). In addition, the most recent evaluation of healthcare service utilisation on a nationwide level showed that there is consistently high utilisation of secondary healthcare services (11). Health policies in Austria aim to redirect patients from higher level care towards primary care services. One of these reforms is the implementation of multidisciplinary primary healthcare units since 2015. By the time of the last ATHIS survey in 2019, there were significant differences in the regional distribution of newly established primary care centres, with most of them located in the state of Styria. However, only 1.37% of the entire Austrian population received care in such facilities at that time (23).

This study aimed to evaluate trends in healthcare utilisation behaviour in relation to different Austrian federal states and population/physician density over time, including regional health policies.

## Methods

This study primarily analysed data from two ATHIS waves (i.e., 2014 and 2019), using self-reported data on healthcare system utilisation as the primary outcome variable. In addition, an ecological study was conducted that treated Austria’s nine federal states as units of observation. The association between aggregated data from the two ATHIS waves and administrative data from the public healthcare system was examined.

### Austrian health interview survey series

The ATHIS series is a nationwide tool for measuring health and health-related parameters, which is conducted by Statistics Austria, the national institute for statistics. The questions in the ATHIS series cover subjective health status, health behaviour, chronic diseases and visits to various levels of health services. Samples were randomly drawn, representing the general population aged over 15 years based on a predefined target number of individuals for each geographical region of Austria to ensure representativeness on a regional basis. The data were then stratified and weighted by various socio-demographic parameters, including sex, age, highest educational level and country of origin. For this analysis, we used data from the 2014 and 2019 surveys due to the lack of the variable ‘degree of urbanisation’ in the 2006/07 dataset for consistency. ATHIS methodology included conducting computer-assisted telephone interviews with survey participants from ATHIS 2014 and computer-assisted personal interviews as well as using a web-based interviewing tool with survey participants from ATHIS 2019. Response rates for the two ATHIS waves (i.e., 2014, 2019) were 40.7 and 50.5%, resulting in net sample sizes of 15,770 and 15,461 individuals, respectively (20–22, 24).

#### Outcome variables for healthcare utilisation

The relevant survey questions for this study were consistent across both the ATHIS 2014 and 2019 waves. Participants were asked if they had visited a GP for office or home visits at least once within the 12 months before the survey to assess their utilisation of primary care services. Similarly, their utilisation of secondary care was evaluated by inquiring about medical specialist visits in ambulatory practices, as well as hospital outpatient and inpatient stays (i.e., day clinics and overnight stays) in the 12 months prior to the survey. Utilisation was assessed regardless of whether consultations were reimbursed by SHI or paid out-of-pocket. The participants’ use of secondary healthcare services without visiting a GP was determined by their ‘yes’ response to the question about utilising secondary care services when there were no visits to a GP in the 12 months prior to the survey. Due to unregulated access and the high utilisation of secondary care, this variable was used as a proxy measure for the direct utilisation of secondary healthcare services.

Based on the Eurostat standard for ‘degree of urbanisation’, regions were classified as having a ‘high’, ‘medium’ or ‘low’ degree of urbanisation according to the ATHIS 2014 and 2019 datasets in reference to urban, suburban and rural regions, respectively. This classification relies on population size, density and the spatial continuity of 1 km^2^ grid cells with similar patterns of urbanisation (25).

#### Socio-demographic factors

Socio-demographics were included in the analysis to further stratify the sample: sex (i.e., male, female), age (i.e., 15–34 years, 35–54 years, 55–74 years and >75 years), highest educational level based on the International Standard Classification of Education (i.e., primary, secondary and tertiary education) and country of birth (i.e., Austria, European Union [EU, including the European Economic Area], Switzerland and the United Kingdom [which was an EU member during the investigated ATHIS waves] and non-EU countries).

### Physician density, population numbers and hospital system indicators

The Austrian Medical Chamber provided data for the number of physicians working in the ambulatory sector in 2014 and 2019, including all physicians working in ambulatory practices (i.e., GPs and specialists), both with and without contracts with SHI companies, broken down by federal state.

The population numbers for the respective Austrian federal states in 2014 and 2019 as well as indicators regarding the hospital system (acute care hospital discharges from inpatient care and health expenditure for public care hospitals, both stratified by federal states) were obtained from Statistics Austria (26, 27).

We assessed the density of SHI-contracted and non-SHI-contracted (private) physicians working in ambulatory practices by determining the number of inhabitants per physician in each federal state, respectively, overall and stratified by GPs and specialists. Furthermore, we calculated the specialist/GP ratio as well as the ratio between SHI-contracted and non-SHI-contracted physicians in the ambulatory sector.

### Statistical analysis

Descriptive analyses using absolute and relative numbers from the ATHIS samples as well as the Austrian Medical Chamber and Statistics Austria data regarding physician and population density were conducted. Subgroup comparisons were performed using cross-tabulations and analysed using Pearson’s chi-squared test.

Two binary logistic regression models were created. Model 1 used the respective health service utilisations as dependent variables, while the year of investigation and Austrian federal states were independent variables. In Step 1 of the analysis, the interaction term between the Austrian federal states and the year of investigation was added to this model to determine changes in health service utilisation over time and across federal states. In Step 2, the model was adjusted for all socio-demographic variables, including the degree of urbanisation, the presence/absence of chronic diseases and the number of chronic diseases as separate control variables to acknowledge this possibly confounding factor.

In Model 2, the respective health service utilisations served as dependent variables again for analysing the ATHIS 2014 and 2019 waves together to identify the differences between federal states and according to the degree of urbanisation. The adjustment variables were the same as for Step 2 of Model 1. The results are presented as odds ratios with 95% confidence intervals (CIs).

Exploratory two-sided Spearman’s rho correlations were calculated to analyse the relationships between the utilisation of healthcare services and the number of inhabitants per ambulatory physician (contracted and non-contracted), the number of inhabitants per GP/specialist, as well as the specialist/GP ratio in the respective Austrian federal states. Hence, a higher number of inhabitants per physician means a lower physician density relative to the population of the respective federal states and vice versa.

All calculations were performed using SPSS Statistics software (v. 30.0; IBM SPSS, Armonk, NY, USA).

### Ethical considerations

The Ethics Committee of the Medical University of Vienna approved the analyses conducted in this study (EC #1654/2023, Amendment 15 December 2024).

## Results

Data from the 31,231 participants in the ATHIS 2014 and 2019 waves were analysed. The highest GP visit rates were observed in the rural state of Burgenland (85.4% in 2019), while the lowest were in Vorarlberg, which is comparatively highly urbanised (74.3% in 2019). Specialist visit rates remained consistent or slightly increased between 2014 and 2019, while outpatient hospital visit rates were inconsistent. Day clinics and overnight hospital stays increased in most federal states between 2014 and 2019, while specialist visits without accompanying GP visits reached their highest point in 2014 in all federal states except Tyrol, which showed slightly higher visit rates in 2019 compared to 2014 (17.0% vs. 16.5%).

Visits of hospital outpatient clinics without a GP followed a similar trend, with highest numbers in 2014 in all federal states except Tyrol, which slightly surpassed its 2014 visit counts in 2019. Patterns of hospital stays without GP visits were consistent with other secondary care visits without accompanying GP visits, both across federal states and over time. In 2019, the highest number of visits of this type was seen in Vorarlberg (18.2%) while the lowest was in Burgenland (7.2%; Table 1).

**Table 1:** Utilisation of different levels of healthcare in Austrian federal states and according to the degree of urbanisation (ATHIS 2014, *n* = 15,770; ATHIS 2019, *n* = 15,461).

	GP visits *n* (%)		Specialist visits *n* (%)		Outpatient visits *n* (%)		Hospital stays *n* (%)		Specialist visits without GP *n* (%)		Outpatient visits without GP *n* (%)		Hospital stays without GP *n* (%)	
	2014	2019	2014	2019	2014	2019	2014	2019	2014	2019	2014	2019	2014	2019
High degree of urbanisation	4,149 (74.9)	3,613 (76.9)	4,210 (76.0)	3,594 (76.5)	1,414 (25.5)	1,369 (29.1)	1,296 (23.4)	1,234 (26.2)	955 (20.4)	649 (18.1)	235 (16.6)	162 (11.8)	168 (13.0)	124 (10.0)
Medium degree of urbanisation	3,230 (76.6)	3,779 (80.4)	3,164 (75.0)	3,556 (75.7)	1,081 (25.6)	1,231 (26.2)	1,009 (23.9)	1,373 (29.2)	636 (18.2)	573 (16.1)	169 (15.6)	129 (10.5)	116 (11.5)	137 (10.0)
Low degree of urbanisation	4,644 (77.3)	4,890 (80.7)	4,355 (72.5)	4,381 (72.3)	1,439 (23.9)	1,543 (25.5)	1,376 (22.9)	1752 (28.9)	840 (17.4)	610 (13.9)	183 (12.7)	140 (9.1)	163 (11.9)	131 (7.5)
*p*	<0.01*	<0.01*	<0.01*	<0.01*	0.07	<0.01*	0.48	<0.01*	<0.01*	<0.01*	<0.01*	0.05	0.52	0.02*
Austria	12,020 (76.2)	12,281 (79.4)	11,730 (74.4)	11,530 (74.6)	3,933 (24.9)	4,143 (26.8)	3,680 (23.3)	4,361 (28.2)	2,200 (18.8)	1831 (15.9)	586 (14.9)	431 (10.4)	448 (12.2)	391 (9.0)
Vienna	2,432 (74.1)	2,554 (78.0)	2,496 (76.0)	2,513 (76.8)	873 (26.6)	904 (27.6)	755 (23.0)	787 (24.0)	544 (21.8)	406 (16.1)	151 (17.3)	88 (9.7)	99 (13.1)	62 (7.9)
Burgenland	421 (78.1)	446 (85.4)	426 (78.9)	396 (75.9)	155 (28.8)	130 (24.9)	143 (26.5)	125 (23.9)	74 (17.4)	35 (8.8)	18 (11.6)	8 (6.2)	12 (8.3)	9 (7.2)
Carinthia	778 (75.1)	787 (79.1)	795 (76.7)	775 (77.9)	250 (24.1)	281 (28.2)	272 (26.3)	323 (32.5)	159 (20.0)	127 (16.4)	37 (14.8)	36 (12.8)	34 (12.5)	29 (9.0)
Lower Austria	2,395 (79.4)	2,421 (82.0)	2,319 (76.9)	2,266 (76.7)	739 (24.5)	717 (24.3)	661 (21.9)	859 (29.1)	363 (15.7)	315 (13.9)	83 (11.2)	66 (9.2)	68 (10.3)	73 (8.5)
Upper Austria	2005 (76.5)	2040 (79.4)	1839 (70.2)	1829 (71.2)	635 (24.2)	722 (28.1)	598 (22.8)	778 (30.3)	335 (18.2)	299 (16.3)	103 (16.2)	87 (12.0)	67 (11.2)	72 (9.3)
Salzburg	734 (74.7)	743 (77.1)	699 (71.1)	702 (72.8)	238 (24.2)	214 (22.2)	240 (24.4)	276 (28.6)	135 (19.3)	125 (17.8)	30 (12.6)	18 (8.5)	23 (9.5)	23 (8.3)
Styria	1736 (76.4)	1760 (80.1)	1710 (75.3)	1,600 (72.8)	567 (25.0)	632 (28.8)	535 (23.5)	687 (31.3)	320 (18.7)	254 (15.9)	95 (16.8)	59 (9.3)	74 (13.8)	58 (8.4)
Tyrol	1,043 (78.1)	1,027 (78.5)	991 (74.2)	959 (73.3)	315 (23.6)	372 (28.4)	324 (24.3)	377 (28.8)	163 (16.5)	163 (17.0)	35 (11.1)	44 (11.8)	35 (10.8)	38 (10.1)
Vorarlberg	476 (69.6)	503 (74.3)	45 (66.5)	490 (72.4)	161 (23.5)	171 (25.3)	152 (22.2)	149 (22.0)	107 (23.5)	107 (21.8)	34 (21.1)	25 (14.6)	36 (23.7)	27 (18.2)
*p*	<0.01*	<0.01*	<0.01*	<0.01*	0.13	<0.01*	0.09	<0.01*	<0.01*	<0.01*	<0.01*	0.09	<0.01*	0.02*

* significance level *p* < 0.05. Secondary care utilisation without GP refers to secondary care visits when there were no GP visits in the observation period. This does not imply causality. GP, general practitioner. Bold values are national level summary statistics.

Descriptive analyses of utilisation patterns, stratified by socio-demographic variables, revealed that female and higher educated participants were more likely to use specialist care, while no consistent trends were observed across federal states.

The utilisation of GPs was significantly higher in the less densely populated regions of Austria (76.9% in highly urbanised regions vs. 80.7% in regions with a low degree of urbanisation in 2019, *p* < 0.01), while these regions showed the opposite utilisation trend for ambulatory specialist visits. In 2019, utilisation rates of this type were 76.5% in highly urbanised regions compared to 72.3% in regions with a low degree of urbanisation (p < 0.01). Visits to secondary care without GP visits were also more frequent in regions of higher urbanisation. These utilisation trends hold true in the data from both the ATHIS 2014 and 2019 waves (Table 1).

Table 2 presents the urbanisation of the respective federal states for the 2014 and 2019 data. The metropolitan region of Vienna was highly urbanised (100% ‘high degree of urbanisation’ in 2014 and 2019), while the lowest degree of urbanisation was seen in Burgenland (80.1% ‘low degree of urbanisation’ in 2019).

**Table 2:** Urbanisation of Austrian federal states according to the absolute and relative numbers of survey participants in ATHIS 2014 and 2019.

	High degree of urbanisation		Medium degree of urbanisation		Low degree of urbanisation	
	2014 *n* (%)	2019 *n* (%)	2014 *n* (%)	2019 *n* (%)	2014 *n* (%)	2019 *n* (%)
Austria	**5,542 (35.1)**	**4,701 (30.4)**	**4,218 (26.7)**	**4,699 (30.4)**	**6,010 (38.1)**	**6,060 (39.2)**
Vienna	3,284 (100)	3,274 (100)	0 (0)	0 (0)	0 (0)	0 (0)
Burgenland	0 (0)	0 (0)	167 (31.0)	104 (19.9)	372 (69.0)	418 (80.1)
Carinthia	200 (19.3)	171 (17.2)	267 (25.8)	223 (22.4)	569 (54.9)	602 (60.4)
Lower Austria	256 (8.5)	0 (0)	1,316 (43.6)	1,363 (46.2)	1,443 (47.9)	1,589 (53.8)
Upper Austria	481 (18.4)	329 (12.8)	940 (35.9)	998 (38.8)	1,199 (45.8)	1,243 (48.4)
Salzburg	306 (31.1)	269 (27.9)	298 (30.3)	284 (29.5)	380 (38.6)	411 (42.6)
Styria	453 (19.9)	474 (21.6)	566 (24.9)	633 (28.8)	1,253 (55.1)	1,089 (49.6)
Tyrol	246 (18.4)	184 (14.1)	415 (31.1)	539 (41.2)	675 (50.5)	586 (44.8)
Vorarlberg	316 (46.2)	0 (0)	249 (36.4)	555 (82.0)	119 (17.4)	122 (18.0)

Sum of relative frequencies may differ from 100% due to rounding (ATHIS 2014, *n* = 15,770; ATHIS 2019, *n* = 15,461). Bold values are national level summary statistics.

Considering the density of physicians across the federal states between 2014 and 2019, the nationwide mean number of inhabitants per ambulatory specialist decreased between 2014 and 2019 (824 inhabitants per specialist in 2019 vs. 873 in 2014), while the mean number of inhabitants per GP increased during this period (Table 3). Table 4 shows a decrease in the density of SHI-contracted physicians between 2014 and 2019, while that of non-SHI-contracted physicians increased. In the same period, acute care hospital discharges dropped for all federal state residents, showing large variation, with the lowest numbers observed in Vienna (237 per 1,000 inhabitants in 2019). Health expenditure for public hospital care per capita increased in all federal states, with Vienna showing the highest expenditure.

**Table 3:** Density of ambulatory GPs and specialists in Austria and Austrian federal states for 2014 and 2019 (both SHI-contracted and non-SHI-contracted).

	Inhabitants per GP		Inhabitants per specialist		Specialist/GP ratio	
	2014	2019	2014	2019	2014	2019
Austria	**M = 1361.7** **(SD 155.0)** **(95% CI: 1242.6–1480.9)**	**M = 1385.6** **(SD 174.0)** **(95% CI: 1251.9–1519.4)**	**M = 873.1** **(SD 171.2)** **(95% CI: 741.6–1004.7)**	**M = 824.0** **(SD 152.8)** **(95% CI: 706.6–941.4)**	**1/0.64**	**1/0.60**
Vienna	1,269	1,272	494*	488**	1/0.39	1/0.38
Burgenland	1,317	1,402	949	863	1/0.72	1/0.62
Carinthia	1,321	1250*	858	842	1/0.65	1/0.67
Lower Austria	1,254	1244*	822	771	1/0.66	1/0.62
Upper Austria	1,283	1,339	1038*	988*	1/0.81	1/0.74
Salzburg	1,323	1,317	761	721	1/0.57	1/0.55
Styria	1,248	1,326	1029*	963*	1/0.82	1/0.73
Tyrol	1534*	1540*	904	847	1/0.59	1/0.55
Vorarlberg	1781**	1781**	932	932	1/0.59	1/0.52

* values below or above 95% CI. ** z-score differs > 2. Values are reported as whole numbers after rounding. GP, general practitioner; M, mean; SD, standard deviation; CI, confidence interval. Bold values are national level summary statistics.

**Table 4:** Density of SHI-contracted and non-SHI-contracted physicians (i.e., ambulatory GPs and specialists) in Austria and Austrian federal states for 2014 and 2019.

	Inhabitants per SHI-contracted physician		Inhabitants per non-SHI-contracted physician		Ratio SHI-contracted/non-SHI-contracted physicians	
	2014	2019	2014	2019	2014	2019
Austria	**M = 1082.8** **(SD 145.7)** **(95% CI: 970.8–1194.8)**	**M = 1119.3** **(SD 100.3)** **(95% CI: 1042.2–1196.5)**	**M = 1049.9** **(SD 249.9)** **(95% CI: 857.8–1241.9)**	**M = 966.0** **(SD 230.6)** **(95% CI: 788.8–1143.3)**	**1/1.11**	**1/1.31**
Vienna	815*	975*	627*	550*	1/1.30	1/1.77
Burgenland	1,187	1232*	1,030	941	1/1.15	1/1.31
Carinthia	1,037	1,049	1,038	959	1/1	1/1.09
Lower Austria	1250*	1,180	820*	795	1/1.52	1/1.48
Upper Austria	1,139	1253*	1,153	1,039	1/0.99	1/1.21
Salzburg	1,092	1,126	860	791	1/1.27	1/1.42
Styria	930*	1003*	1430*	1255*	1/0.65	1/0.80
Tyrol	1,041	1,070	1244*	1,111	1/0.84	1/0.96
Vorarlberg	1254*	1,185	1245*	1253*	1/1.01	1/0.95

	Acute care hospital discharges from inpatient care per 1,000 inhabitants stratified by state of residence		Total healthcare expenditure for federal state public hospitals per capita and year in Euros	
	2014	2019	2014	2019
Austria	**M = 311.9** **(SD 19.7)** **(95% CI: 296.7–327.0)**	**M = 265.8** **(SD 14.0)** **(95% CI: 255.0–276.6)**	**M = 1,131** **(SD 226.7)** **(95% CI: 957.1–1305.7)**	**M = 1,340** **(SD 253.7)** **(95% CI: 1145.4–1535.5)**
Vienna	290*	237*	1604**	1869**
Burgenland	331*	257	747*	929*
Carinthia	325	281*	1,241	1,427
Lower Austria	287*	259	1,024	1,198
Upper Austria	337*	285*	1,191	1,440
Salzburg	333*	264	1,151	1,421
Styria	302	268	1,076	1,243
Tyrol	307	268	1,040	1,302
Vorarlberg	296*	271	1,110	1,236

Hospital system indicators in Austria and Austrian federal states for 2014 and 2019. * values below or above 95% CI. ** z-score differs > 2. Values are reported as whole numbers after rounding. M, mean; SD, standard deviation; CI, confidence interval. Bold values are national level summary statistics.

The results of the multivariable regression analyses are presented in Table 4. In Model 1, the interaction between federal states and time of observation (2014 vs. 2019) showed significance for the utilisation of several health services (Table 5). In Model 2, the differences in healthcare service utilisation between the federal states and according to the degree of urbanisation were examined for both survey years. The results aligned with the descriptive statistics findings after adjustment for socio-demographic variables. GP visits were more frequent in regions with a low degree of urbanisation compared to highly urbanised regions (OR = 1.22, 95% CI 1.11–1.34, *p* < 0.01), whereas the inverse utilisation pattern was observed for specialist visits in this region (OR = 0.79, 95% CI 0.72–0.86, *p* < 0.01). This led to incremental utilisation tendencies for primary and secondary healthcare services (both with and without a GP visit) according to the degree of urbanisation (Tables 6, 7).

**Table 5:** Logistic regression analysis for health service utilisation according to ATHIS 2014 and 2019 (Model 1, *n* = 31,231).

Step 1 (unadjusted)		Step 2 (adjusted)	
Outcome variables (dependent)	*p*	Outcome variables (dependent)	*p*
GP visits	0.44	GP visits	0.34
Specialist visits	0.12	Specialist visits	0.01*
Outpatient visits	0.01*	Outpatient visits	0.01*
Hospital stays	<0.01*	Hospital stays	<0.01*
Specialist visits without GP	0.02*	Specialist visits without GP	0.02*
Outpatient visits without GP	0.16	Outpatient visits without GP	0.21
Hospital stays without GP	0.69	Hospital stays without GP	0.83

*p*-values for the interaction term ‘federal state*year of investigation’. Step 1 (unadjusted): Independent variables: federal state, year of investigation (i.e., 2014, 2019). Step 2 (adjusted): Independent variables: federal state, year of investigation (i.e., 2014, 2019) + degree of urbanisation, socio-demographic variables (i.e., sex, age, highest educational level, birthplace), presence of chronic diseases (i.e., yes/no), number of chronic diseases. * significance level *p* < 0.05. Secondary care utilisation without GP refers to secondary care visits when there were no GP visits in the observation period. This does not imply causality. GP, general practitioner. Bold values are national level summary statistics.

**Table 6:** Logistic regression analysis for health service utilisation according to ATHIS 2014 and 2019 (Model 2, *n* = 31,231).

Federal states	GP visits				Specialist visits				Outpatient visits				Hospital stays			
	OR	95% CI		*p*	OR	95% CI		*p*	OR	95% CI		*p*	OR	95% CI		*p*
Vienna	1.00				1.00				1.00				1.00			
Burgenland	1.18	0.97	1.42	0.10	1.15	0.96	1.39	0.14	1.05	0.89	1.25	0.55	1.07	0.90	1.28	0.43
Carinthia	0.96	0.83	1.10	0.56	1.14	0.99	1.32	0.07	1.05	0.92	1.20	0.49	1.39	1.22	1.59	<0.01*
Lower Austria	1.11	0.98	1.25	0.10	1.07	0.95	1.21	0.25	0.90	0.81	1.01	0.08	1.05	0.94	1.19	0.37
Upper Austria	1.00	0.89	1.13	0.95	0.80	0.71	0.90	<0.01*	1.03	0.92	1.15	0.62	1.19	1.07	1.33	<0.01*
Salzburg	0.96	0.84	1.10	0.54	0.86	0.75	0.99	0.03*	0.91	0.79	1.04	0.15	1.26	1.10	1.44	<0.01*
Styria	1.00	0.89	1.13	0.95	0.94	0.83	1.06	0.30	1.05	0.94	1.17	0.42	1.22	1.09	1.37	<0.01*
Tyrol	1.06	0.93	1.21	0.40	0.97	0.85	1.10	0.62	1.06	0.93	1.20	0.39	1.25	1.10	1.42	<0.01*
Vorarlberg	0.77	0.66	0.89	<0.01*	0.75	0.64	0.87	<0.01*	0.95	0.82	1.11	0.55	0.98	0.83	1.15	0.79
Degree of urbanisation																
High	1.00				1.00								1.00			
Medium	1.18	1.08	1.30	<0.01*	0.95	0.86	1.04	0.26	0.95	0.86	1.04	0.22	1.04	0.95	1.14	0.38
Low	1.22	1.11	1.34	<0.01*	0.79	0.72	0.86	<0.01*	0.87	0.80	0.95	<0.01*	0.99	0.90	1.08	0.76

Adjusted for socio-demographic variables (i.e., sex, age, highest educational level, birthplace), presence of chronic diseases (i.e., yes/no), number of chronic diseases. * significance level *p* < 0.05. GP, general practitioner; OR, odds ratio; CI, confidence interval. Bold values are national level summary statistics.

**Table 7:** Logistic regression analysis for health service utilisation according to ATHIS 2014 and 2019 (Model 2, *n* = 31,231).

Federal states	Specialist visits without GP				Outpatient visits without GP				Hospital stays without GP			
	OR	95% CI		*p*	OR	95% CI		*p*	OR	95% CI		*p*
Vienna	1.00				1.00				1.00			
Burgenland	0.84	0.66	1.07	0.16	1.00	0.62	1.61	0.99	0.99	0.58	1.68	0.96
Carinthia	1.12	0.94	1.33	0.19	1.36	0.98	1.88	0.07	1.25	0.88	1.79	0.21
Lower Austria	0.94	0.81	1.09	0.43	1.07	0.80	1.43	0.67	1.19	0.86	1.64	0.29
Upper Austria	1.07	0.93	1.23	0.36	1.41	1.08	1.83	0.01*	1.19	0.89	1.61	0.24
Salzburg	1.06	0.90	1.26	0.48	0.87	0.61	1.24	0.45	0.89	0.61	1.30	0.55
Styria	1.10	0.95	1.26	0.21	1.29	0.99	1.68	0.06	1.25	0.93	1.69	0.14
Tyrol	0.98	0.83	1.15	0.77	1.09	0.79	1.50	0.59	1.14	0.81	1.61	0.46
Vorarlberg	1.38	1.14	1.67	<0.01*	1.64	1.15	2.33	0.01*	2.58	1.78	3.74	<0.01*
Degree of urbanisation												
High	1.00				1.00				1.00			
Medium	0.83	0.73	0.93	<0.01*	0.77	0.62	0.96	**0.02**	0.77	0.61	0.98	0.03*
Low	0.75	0.66	0.84	<0.01*	0.63	0.51	0.79	<0.01*	0.70	0.55	0.88	<0.01*

Adjusted for socio-demographic variables (i.e., sex, age, highest educational level, birthplace), presence of chronic diseases (i.e., yes/no), number of chronic diseases. * significance level *p* < 0.05. Secondary care utilisation without GP refers to secondary care visits when there were no GP visits in the observation period. This does not imply causality. GP, general practitioner; OR, odds ratio; CI, confidence interval. Bold values are national level summary statistics.

**Figure 1:**
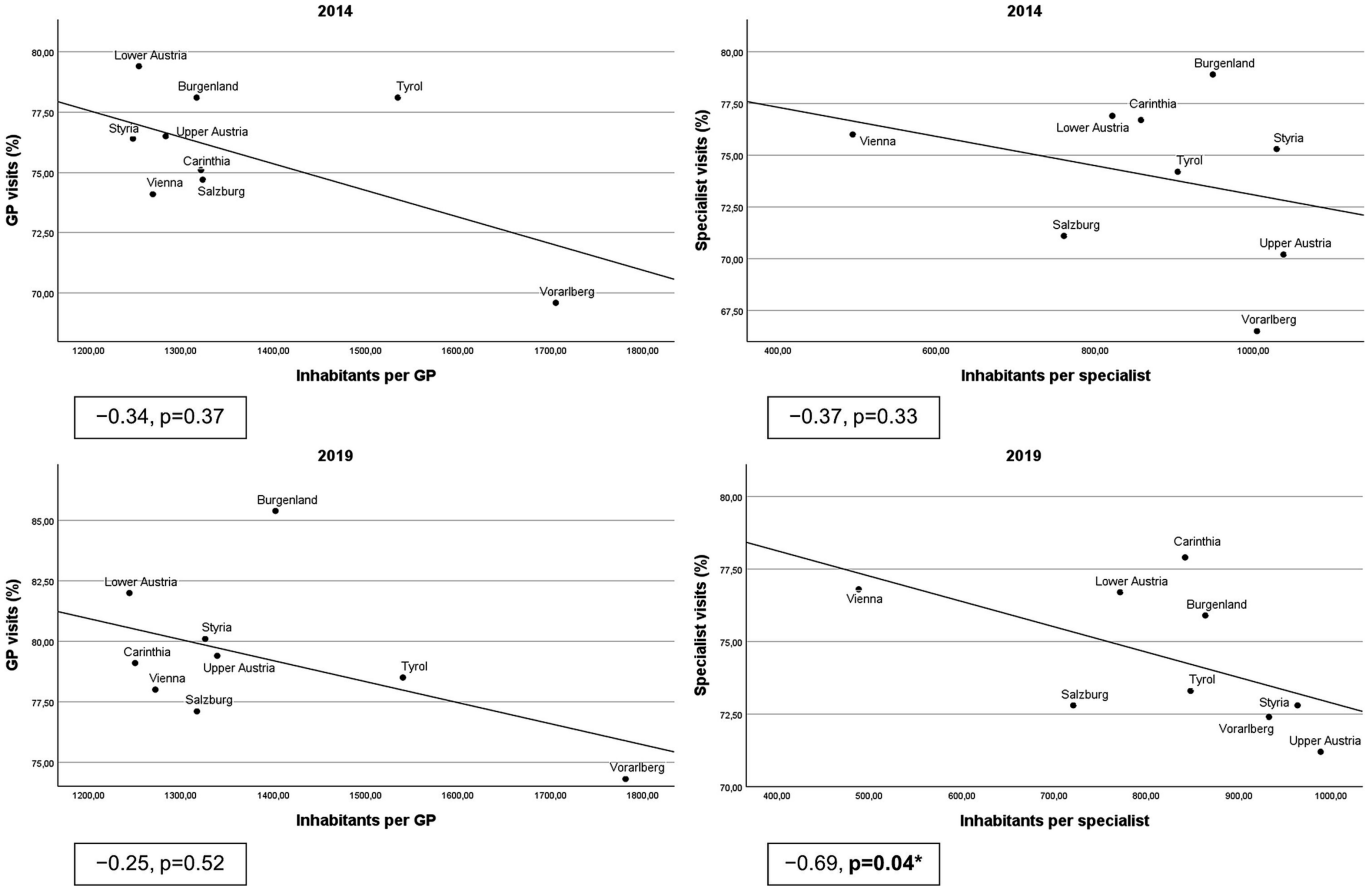
Spearman’s rho correlations of health service utilisation in the ATHIS 2014 and 2019 waves in relation to physician density. * significance level *p* < 0.05. GP, general practitioner.

Exploratory correlation analyses showed an associative trend between the density and utilisation of GPs in the respective federal states, however, not showing significance. 2019 data revealed a negative correlation between the number of inhabitants per ambulatory specialist and utilisation of specialists (Spearman’s rho correlation coefficient: −0.69, *p* = 0.04; Figure 1). Further correlation analyses assessing ambulatory physician density, stratified by the contractual setting they work in (SHI-contracted vs. non-contracted), and healthcare utilisation in the respective federal states did not reveal any significant patterns.

## Discussion

Considering the significant differences in both physician and population density, this study explored the variations in the utilisation of healthcare services across the nine Austrian federal states over a 5-year period. It is remarkable that the overall density of GPs nationwide remains lower than that of specialists. In particular, specialist density increased between 2014 and 2019, while GP density decreased. The high number of specialists can be attributed to the growing trend towards the utilisation of private healthcare services due to the long waiting times in public healthcare. However, private specialist practices only accounted for approx. 15% of public healthcare provision in Austria in 2023, whereas private GP practices contributed even less than 2% (28). In addition, Austria is also experiencing a wave of GPs retiring from general practice.

While Austria’s overall physician density and healthcare spending are among the highest among European countries, waiting times for visits to specialists under the SHI programme still saw a significant increase between 2012 and 2024 (29, 30).

The utilisation of GP services was highest in the rural state of Burgenland, as shown by regression analyses on the degree of urbanisation, which highlighted the differences in utilisation between regions of different degrees of urbanisation. The utilisation of secondary care (both with and without GP visits) gradually increased in regions with higher populations, while GP visits showed an opposite incremental trend.

The 2016 SHI data for the number of individual health service visits also showed that high specialist utilisation is associated with lower GP visit counts (31). For example, Vorarlberg is highly urbanised compared with other Austrian federal states and has the lowest GP density and GP visits. However, the overall density of physicians in a federal state may not necessarily be directly associated with population density, as there may be an uneven distribution (19, 32). The correlation analyses for the 2019 data showed that the number of ambulatory specialist visits is correlated with specialist density in federal states. Overall, utilisation trends may indicate that the availability of healthcare services might be a major driver of their utilisation.

The regression analyses in Model 1 revealed differences in healthcare service utilisation across Austrian federal states and over time. However, adjusting for socio-demographic variables did not lead to major changes in outcomes, which leads to the assumption that the high variability in utilisation among federal states can only be partly explained by demographic determinants, such as age or educational level. This may be due to different health policies among Austrian states, particularly in the provision of hospital care. Interestingly, the largest differences in health service utilisation across the federal states were seen in hospitalisations (i.e., Model 2). The self-reported utilisation of inpatient hospital care remained at a high level in all federal states, while acute care hospital discharge statistics showed a decrease during the time of investigation. Reasons for that could be planned hospitalisations or a reporting bias. Moreover, up from 2019 a new renumeration system was introduced in outpatient hospital care (‘LKF ambulant’ = ‘Leistungsorientierte Krankenanstaltenfinanzierung ambulant’, performance-based outpatient hospital financing system), incentivising outpatient rather than inpatient hospital care, which may have impacted hospitalisations (33). The number and specialisation of hospitals vary across the federal states, as do the organisational features of out-of-hours medical services and the distribution of healthcare provision in the ambulatory setting (e.g., child and adolescent psychiatry or ambulatory cataract surgeries), what is reflected by the large variation in public-funded acute care hospital discharges across the federal states, with the lowest numbers observed in Vienna. This may be partly explained by the high ambulatory physician density of the city. Conversely, health expenditure for public hospital care was highest in Vienna, likely due to the large number of specialised and therefore cost-intensive clinics. However, conclusions must be drawn with caution since governance and financing structures of the Austrian healthcare system are very complex. There were also differences seen in cancer incidence rates, healthy life years, duration of hospitalisation and patient-related factors, such as polypharmacy and potentially inappropriate medication rates across the federal states (34, 35).

The nationwide trend towards increased utilisation of secondary care was also evident at the federal state level (11). Recent data from Austrian SHI companies revealed that 40–50% of patients make initial contact with the healthcare system at the secondary care level, which is exceptionally high internationally (8, 13, 36). The growing number of specialists not contracted with SHI has led to a shift in the specialist/GP ratio towards specialist care with large variations among the federal states. For example, Vienna has by far the highest number of specialists relative to GPs. Surprisingly, the number of specialist visits in Vienna, both with and without a GP visit, did not exceed the corresponding visit rates in other federal states. This finding contrasts with our observation that the ‘supply’ of healthcare facilities (i.e., system level) stimulates ‘demand’ (i.e., patient level) and consequently increases service utilisation. However, it should be noted that several predominantly small practices without SHI contracts in Vienna are run by physicians, in addition to their hospital duties. Therefore, many of these doctors might not support public healthcare solely via operating these practices, as previously indicated (37). In contrast, there are currently several vacant SHI positions in both primary and secondary care for doctors in almost all Austrian federal states (38, 39). There is also an ongoing debate about whether patients from other federal states should travel to Vienna to receive treatment, as local hospitals are already at capacity in providing care to residents. Moreover, some political forces in Austria call for private physicians to be obliged to treat SHI patients to help reduce waiting times in public healthcare.

Patient mobility is closely linked to commuting for work and education from predominantly rural places of residence to urban regions, where individuals might also utilise healthcare services. This may particularly apply to rural federal states, such as Burgenland. However, commuting patterns could not be captured from our data. Given different degrees of urbanisation, healthcare utilisation in the context of commuting from one federal state to another may be subject of future research.

A scoping review investigating higher income countries showed that rural communities utilised fewer health services overall, both in primary and secondary care, mainly due to access barriers, but this may not fully apply to Austria due to unregulated access (40). Therefore, when contextualising our findings from an international perspective, only countries with similar healthcare structures, including unregulated access and a specialist-driven system such as those of the USA and Germany, come into focus. A German study showed that regional variations in utilisation can be largely explained by ‘demand’ factors, such as demographic and socio-economic characteristics, rather than ‘supply’ factors, such as the number and specialisation of regional ambulatory physicians (41). Variations in ‘demand’ factors were reported to be larger in Germany than in the USA, which was attributed to their strong regional identities (42). Considering the historical background, this variation may also be applicable to Austrian federal states and may account for the differences in utilisation behaviour. Swedish data also emphasise demand-side factors for regional variations in healthcare utilisation, while a large part of the variation was assigned to unobserved variables (43).

According to this study, both system- and patient-related parameters may affect utilisation tendencies. Furthermore, how the healthcare reform process in Austria affects regional policies must be confirmed because the overall health governance structures, including unregulated access to all levels of care, have remained unchanged over time. In Austrian federal states, it is necessary to address both ‘demand’ and ‘supply’ factors to properly allocate patients to the appropriate level of care, which will help avoid time- and labour-consuming and therefore more expensive higher-level healthcare utilisation. Regional health assessments can identify population characteristics and health needs. Considering the high proportion of older adults in rural regions, mobile homecare services, such as the ‘community nursing’ project, might prevent unplanned hospitalisations together with telehealth support (44). Furthermore, policy changes in primary care must account for local accessibility of health services in the respective federal states, which may not be entirely addressed by establishing healthcare centres in urbanised areas.

### Strengths and limitations

The main strengths of this study include the large sample size and methodological consistency across the ATHIS series that included the same questions relevant to this study, trained interview teams, random participant selection and data weighting. However, this study has some notable limitations. The retrospective descriptive cross-sectional study design with different participants in the ATHIS waves only allows for hypotheses to be generated on utilisation tendencies rather than inferring causal relationships. Intra-individual changes over time cannot be captured. The data only covered a 12-month time period of primary and secondary healthcare visits, with the most recent survey data available dating back to 2019. Hence, it is not possible to determine which level of healthcare services was first utilised, how often, if the care level was appropriate or not, and if the utilisation was SHI-funded or paid out-of-pocket. Of note, utilisation patterns, such as planned recurrent specialist visits, could not be derived from the dataset. Therefore, secondary care utilisation without GP visits in some cases may also reflect appropriate healthcare utilisation.

Additionally, the data were self-reported and not retrieved from administrative records, which might have introduced a response bias. Response rates were in the expected range for survey studies, however varied by 9.8% across the two survey waves. Interviews were primarily conducted in German language, which may have led to a selection bias by underrepresenting migrant populations. In addition, comparing the respective Austrian federal states might be challenging because of their various socio-economic and geographical conditions, including differences in population density, as well as unobserved parameters that may influence healthcare utilisation. It should be noted that the variable definition (Eurostat) for the different degrees of urbanisation changed between 2014 and 2019, which led to a decrease in the number of urbanised regions. However, there were no major demographic changes observed in terms of increased rural–urban migration during the observation period, except a growing number of individuals born abroad, who reside in Austria, similar to trends across many Central European countries (45). Moreover, the exploratory nature of the correlation analyses with the nine federal states as observational units should be considered. These factors contributed to the study’s overall limited explanatory power. Future studies on similar research topics should involve a multilevel and mixed-method approach to account for the multidimensional nature of healthcare utilisation.

## Conclusion

This cross-sectional series investigated healthcare utilisation across Austrian federal states over time as well as the association between the degree of urbanisation and the utilisation of health services. There were higher rates of GP visits and lower rates of secondary ambulatory care visits in less densely populated regions and vice versa for urban regions. The findings for the respective federal states were mixed due to regional differences in their health policies and socio-demographic parameters. Overall physician density was associated with trends in the utilisation of GPs and specialists in ambulatory care. However, this association did not hold when stratified by SHI- and non-SHI-contracted health services. The utilisation of inpatient hospital care remained at a high level, while acute care hospital discharge statistics showed a decrease over time. The nationwide tendency towards higher secondary care utilisation was also evident at the federal state level. Hence, national health reforms in Austria, particularly in primary care, must consider both federal health policies and regional population characteristics.

## Data Availability

The data analysed in this study is subject to the following licenses/restrictions: this study analysed data from the Austrian Health Interview Survey series from the Austrian national statistics agency. Data is available from that agency. Requests to access these datasets should be directed to Statistik Austria, https://www.statistik.at/services/tools/serviceangebote/publikationen/detail/848.
